# Opening Exercises of the Southern Medical College

**Published:** 1884-10-20

**Authors:** 


### EDITORIALS AND MISCELLANEOUS.

### OPENING EXERCISES OF THE SOUTHERN MEDICAL COLLEGE.

The introductory lecture of Prof. Nicolson in the SOUTHERN MEDICAL COLLEGE, of Atlanta, was able and appropriate to the occasion. Quite a large and intelligent class of young gentlemen were present, and a goodly number of citizens, including ladies and professional gentlemen. The prospects of the school are flattering, and it is evident that the members of the faculty are in earnest in their expressed determination to make Atlanta a great medical centre. It is believed that the equipment of this institution is in every respect unsurpassed by any institution in the whole country, and that it is destined to hold a very high rank among the foremost schools on the continent. The sympathies of the profession throughout the Southern States are rapidly turning toward this school, being struck by its rapid development, its high position in respect to education and the ethics, and the remarkable energy and ability of the faculty.

